# A large parosteal ossifying lipoma of lower limb encircling the femur

**DOI:** 10.1186/1755-7682-7-5

**Published:** 2014-01-16

**Authors:** Atif Ali Hashmi, Babar Malik, Muhammad Muzzammil Edhi, Naveen Faridi, Muhammad Ashraful

**Affiliations:** 1 Department of histopathology, Liaquat national hospital and medical college, Karachi, Pakistan; 2 Department of medical oncology, Sindh institute of urology and transplantation, Karachi, Pakistan; 3 Liaquat National hospital and medical college, Karachi, Pakistan; 4 Dhaka medical college, Dhaka, Bangladesh

## Abstract

**Introduction:**

Lipoma is a benign soft tissue neoplasm that may contain mesenchymal elements, as a result of metaplastic process. Ossification in benign and malignant soft tissue tumors can also manifest due to metaplastic process.

**Case presentation:**

A 45 year old woman presented with a large thigh mass. The mass was developed one and a half year ago which insidiously increased in size and was associated with movement restriction. Radiological findings revealed soft tissue neoplasm on antero-medial aspect of thigh encircling the femur and displacing adjacent muscles. Fine trabeculations were seen in neoplasm suggestive of ossification. Excision of the mass was performed and histopathology revealed adipocytes with mature bony trabeculae possessing prominent osteoblastic rimming suggestive of ossifying lipoma.

**Conclusion:**

It is important to recognize this variant of lipoma as it is associated with a better clinical outcome in contrast to most of the deep seated soft tissue neoplasms. Secondly it should also be differentiated from myositis ossificans and heterologous differentiation in other soft tissue neoplasms. We suggest an algorithmic approach to the diagnosis of ossifying soft tissue neoplasms histopathologically. Mature bony trabeculae with prominent osteoblastic rimming in a soft tissue lesion are due to a metaplastic process and should not be confused with osteosarcoma.

## Introduction

Lipoma is a benign tumor of mature adipose tissue mostly witnessed in superficial locations of the body. A variety of mesenchymal elements including smooth muscles, skeletal muscle, cartilage and bone may develop in lipoma, some of them as a result of metaplastic process [1]. Lipomas are prevelant in adults ranging from 40 to 60 years of age. Lipoma does not have genetic predisposition however genetic studies carried out in mice have shown correlation with HMG I-C gene and lipoma development [2]. HMG I-C gene belongs to non histone chromosomal high mobility group protein family, associated with adipogenesis and mesenchymal differentiation. Gene silencing studies have depicted its role in diet induced obesity.

Clinical presentation of ossifying lipoma depends upon the site of origin. Parosteal ossifying lipoma usually are in intimate contact with periostium of long bones. They may remain completely asymptomatic or erode adjacent bone and joint spaces producing symptoms of degenerative joint disease. While in other cases, it may compress adjacent nerves and present as a painful lesion especially if it occurs wrists where nerve bundles are running. Although variations seen in lipomas, don’t change the course of the disease significantly and therefore are of little prognostic significance, however it is very important to recognize these variants, as they may be clinically confused with other benign and malignant processes. Of these variants, ossifying lipoma is the least commonly recognized entity. Ossification in soft tissue neoplasms is not unique to lipomas and can occur in other benign and malignant processes like myositis ossificans, dedifferentiated liposarcoma, fibromyxoid sarcoma, synovial sarcoma and extraskeletal osteosarcoma, therefore high index of suspicion is needed to evaluate ossifying soft tissue neoplasms [3,4]. To date there is no evidence of malignant transformation of ossifying lipoma. There are a few case reports of ossifying liposarcoma [5] but they may well represent denovo sarcoma rather than originating from a benign ossifying lipoma.

## Case presentation

A 45 year old healthy woman presented with a thigh mass for one and a half year. The mass insidiously increased in size with no associated pain; however it was associated with difficulty in walking and maintaining upright posture during sitting. There were no associated co-morbids. The clinical suspicion was that of a malignant soft tissue neoplasm. Preoperative findings revealed that the mass was well circumscribed peripherally and not adherent to adjacent muscles, however it was firmly adherent to the underlying bone. Trucut biopsy of the mass was done and the diagnosis was suggestive of a lipomatous tumor.

### Radiology

**Figure 1:**
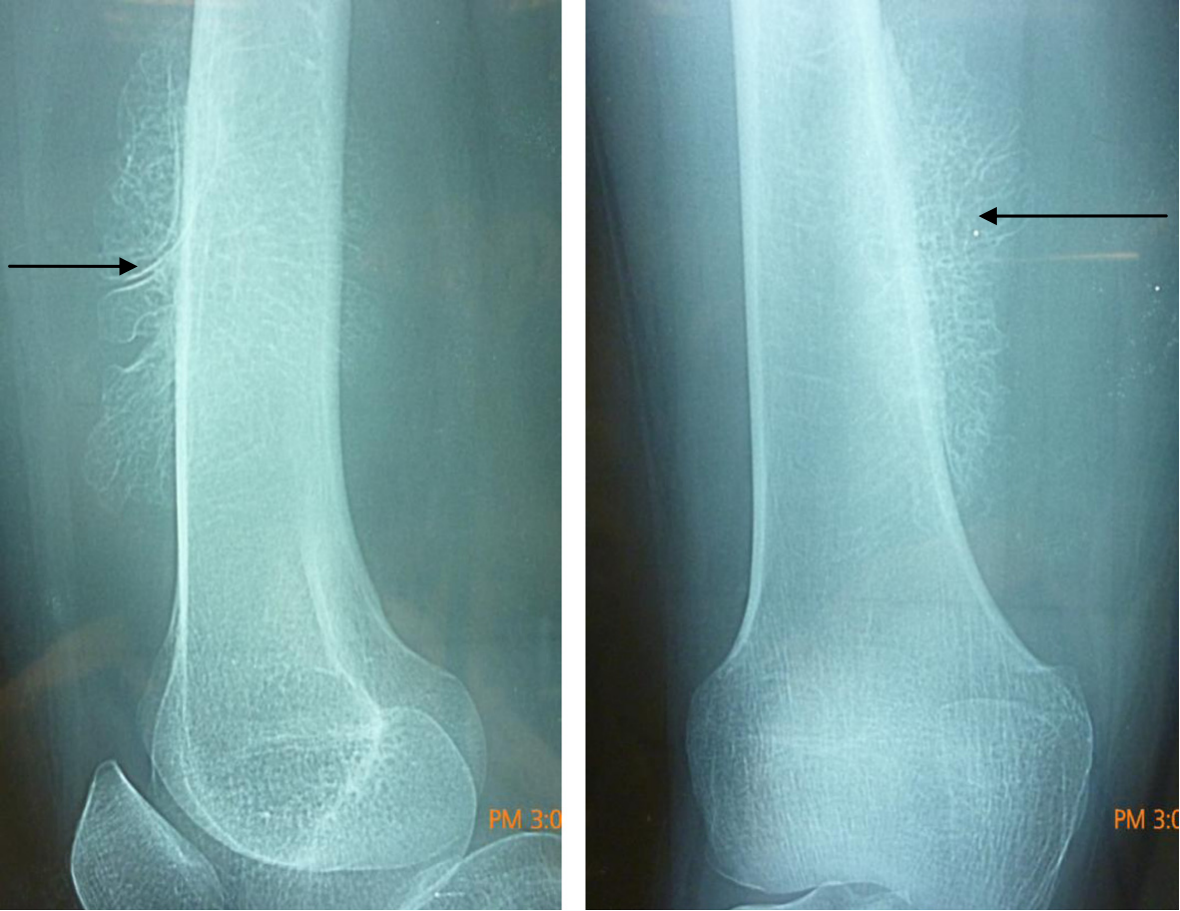
**Antero-medial aspect of x-rays showing an ossified soft tissue mass.** The arrow shows the attachment of the ossification to the femur.

X-ray revealed a well defined radiolucent lesion in the soft tissues of lower one-third of the thigh along antero-medial aspect of the femur. It shows areas of ossification with fine trabeculations (Figure 1).

**Figure 2:**
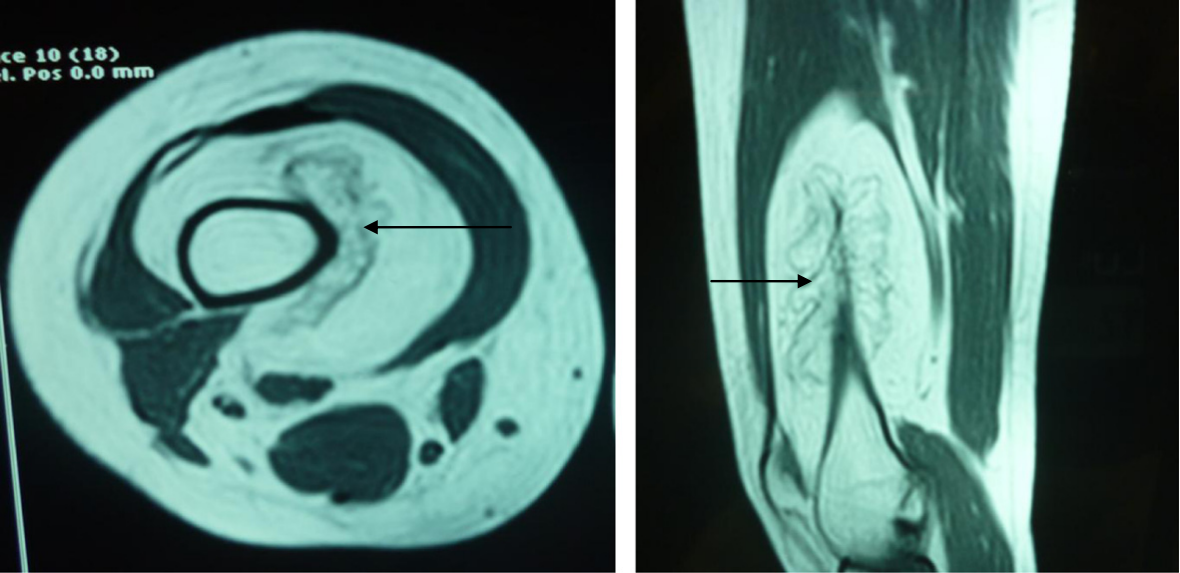
MRI (Axial T1 (3.A) and T2 (3.B) weighted) of the lesion showing a well-defined ossified mass, along the anteromedial aspect of long shaft of femur.

MRI revealed a well defined lesion with signal intensity suggestive of fat along the distal shaft of the femur, predominantly along its antero-medial aspect. It measures approximately 8 cm × 6.5 cm × 14 cm (APx TSx CC) displacing and wrapping the adjacent muscles. It shows lobulated and trabeculated areas which appear low on pulse sequences, most likely representing areas of ossification with no evidence of erosion of underlying bone (Figure 2).

### Histopathology

**Figure 3:**
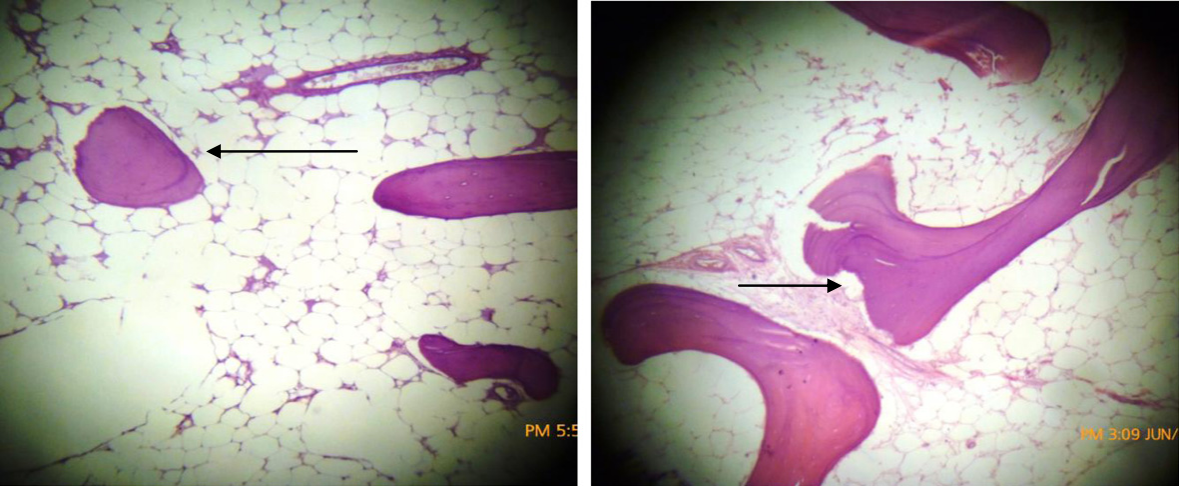
H & E sections showing adiopose tissue with interspersed mature bone trabeculae.

Hematoxylin and eosin sections of the tumor revealed mature adipocytes having eccentric nuclei devoid of atypia and large clear cytoplasmic vacuoles intermixed with adipocytes, possessing mature bony trabeculae with prominent osteoblastic rimming. Zonation pattern was absent. Intervening fibrous septa were appreciated with absence of lipoblast, atypical cells in soft tissue component of the tumor and fibroblastic type stroma (Figure 3).

## Discussion

Lipomatous tumors range from benign lipomas to dedifferentiated liposarcomas. Lipomas are slow growing tumors which can achieve large sizes if left un-noticed as seen in our case and requires clinical attention primarily due to cosmetic reasons. Besides angiolipoma, they are not usually painful unless impinging on a nerve. Parosteal ossifying lipoma can present clinically with dual histogenesis for osteochondromatous component, however in our case H & E stain depicts adipocytes with bony trabeculae while no intervening cartilage.

Ossifying liopmas are described at various sites including thigh [6,7] hand [8,9], groin [10], thorax [11], retroperitonium [12], shoulder [13], intracranial [14], spinal [15], oral [16] and parapharyngeal [17,18] locations. Ossifying lipoma usually occur in parosteal (adjacent to bone) or intraosseous locations, however intramuscular ossifying lipomas have also been reported [19]. To our knowledge, not a single case of ossifying lipoma has been reported from Pakistan, this is not well recognized clinical entity in this part of the world.

Pathogenesis of ossifying lipoma is largely theoretical and considered to be a metaplastic process. Ossifying lipoma can sometimes be wrongly interpreted as myositis ossificans when there is no association with the underlying bone, therefore high index of suspicion is prerequisite in analyzing benign ossifying soft tissue lesions so that distinction between myositis ossificans and ossifying lipomas can be ascertained. Ossifying lipoma should also be differentiated from secondary bone formation as a result of heterologous differentiation in other soft tissue neoplasms such as dedifferentiated liposarcoma and osteosarcoma.

Therefore we recommend an algorithmic approach in assessing ossifying soft tissue neoplasms histopathologically. First, the soft tissue component should be analyzed for growth pattern, anaplasia and mitotic activity, followed by assessment of bony component. When the soft tissue component is malignant with mature bony trabeculae having osteoblastic rimming, the diagnosis will depend upon the growth pattern and immunohistochemical profile of soft tissue compartment and the bone is formed as a result of metaplastic process as seen in dedifferentiated liposarcoma, chondrosarcoma, synovial sarcoma, carcinosarcoma. A benign soft tissue component with mature bone will also point towards a metaplastic process. In such circumstances a fibroblastic stroma will lead to a diagnosis of myositis ossificans while a lipomatous soft tissue compartment should lead to a diagnosis of ossifying lipoma. A malignant/ immature bony component without osteoblastic rimming, formed directly by tumor cells should lead to a consideration of osteosarcoma. It is important to recognize this variant of lipoma because it is usually deep seated and adherent to periosteum, however in contrast ossifying lipoma is associated with a better clinical outcome at this location.

Treatment modalities of periosteal lipoma depends upon the circumstances that mass presents, it needs to be removed in cases where it is painful or restricts movements. In our case periosteal lipoma was surgically removed by simple excision and no evidence of recurrence was observed.

## Conclusion

Ossifying lipoma is a rare variant of lipoma, diagnosis of which requires high index of suspicion as it can sometimes be wrongly interpreted as myositis ossificans or heterologous differentiation in other soft tissue neoplasms. We suggest an algorithmic approach to the diagnosis of ossifying soft tissue neoplasms. Mature bony trabeculae with prominent osteoblastic rimming is almost always due to metaplastic process and should not be confused with osteosarcoma.

## Consent

Patient has given their informed consent for the case report to be published.

## Competing interests

The authors declare that they have no competing interests.

## Author’s contributions

AAH: main author of manuscript, have made substantial contributions to conception, design and acquisition of data. BM: involved in drafting the manuscript and revising it critically for important intellectual content. MME: involved in drafting the manuscript and revising it critically for important intellectual content. NF: have given final approval of the version to be published. MA: have given final approval of the version to be published. All authors read and approved the final manuscript.
